# Foreign Bodies in the Anterior Chamber of the Eye

**Published:** 1882-04

**Authors:** William Oliver Moore

**Affiliations:** Professor Comparative Ophthalmology, Columbia Veterinary College


### ART. VIII.—FOREIGN BODIES IN THE ANTERIOR CHAMBER OF THE EYE.

BY WILLIAM OLIVER MOORE, M.D.,
Professor Comparative Ophthalmology, Columbia Veterinary College.

The eye, though so well protected by its natural coverings, the upper and lower eyelids, and, in some animals, by the nictitating membrane, is often taken by surprise, and foreign bodies enter its cavity.

Most foreign bodies enter the anterior chamber from without by injury, yet a few find their way thither through the blood, and then manifest their nature.

We will first speak of those which enter from without by injury inflicted upon the organ. The substances thus found in the anterior chamber are very numerous. The following are among the more common: Steel, iron, glass, gun-cap fragments, stone, wood, powder, and many others.

Such substances may pierce the cornea without passing through it, and project into the chamber. They may enter the latter completely, and lie in the bottom of it, or they may stick in the iris. The removal of the foreign body which seems to be urgently indicated is not always easy, especially in the inflamed state of the eye, and requires manipulations likely to increase the inflammation and lead to defect of vision. So, often we have to choose between the two evils—continued inflammation from the presence of the foreign substance, or danger to the eye from the operation to remove it.

As a general rule, however, it is best to remove them as soon as possible with the least amount of violence.

In those instances where the substance has not entirely passed through the cornea, but is still held partly by it, one end lying in the anterior chamber, it is often difficult to select a method of procedure. In these cases attempts to grasp the body by forceps from without will often force it into the chamber, thus complicating the situation. In such a case, I would advise that a flat-bladed knife be made to pass through the sclero-corneal junction into the chamber, and passed immediately behind the inner point of the body, thus keeping it in position; then cut down through the cornea at the point of entrance of the substance; reaching it, grasp by fine-toothed forceps, and remove it; then gently withdraw the knife from the chamber, which has been used simply to steady the body.

In those cases where it has entered the chamber and is lying at its bottom, and where iron or steel are entangled in the tissue of the iris, we may resort to the following procedure: Take a reamer-knife and make a small incision at the sclero-corneal junction opposite the foreign body, allowing the instrument to pass into the chamber; then gently withdraw it, at the same time making slight pressure on the lower lip of the incision, thus favoring the escape of the aqueous humor, and with it, in most cases, the foreign body. If it should not come away with the aqueous, forceps may be introduced through the incision, and the body grasped, or, in the case of its being iron or steel lying loose, or entangled in the iris, a magnet may be introduced, and the foreign bodies be thus removed. These magnets are now made in very practical shape for this purpose, that by Dr. Gruening being the most convenient. In some of the cases where the body is entangled in the iris other than iron or steel, in which it cannot be removed alone, it is perfectly proper to do an iridectomy, including the body in the portion of iris removed.

As to the substances found in the anterior chamber which come from the blood, we will first speak of the products of inflammation sometimes found here, and then of those entozoa which make this their abode.

Hypopyon or pus, in the anterior chamber, is a frequent symptom of severe keratitis or iritis. The pus is found always at the bottom, and has a crescentric-shaped appearance. If the position of the head be changed for any length of time, the pus will gravitate to the most dependent portion of the chamber; thus we are able to distinguish between free pus in the aqueous and that infiltrated in the corneal substance. In those instances where the hypopyon is not absorbed by medicinal measures, such as bathing with hot water, instillation of atropine, and the like, resort may be had to surgical measures, namely, paracentesis corneæ, thus evacuating the pus. In most cases the warm applications and atropia will cause absorption. Hyphæmia, or blood in the anterior chamber, may arise either from traumatism or from inflammatory action; in either case the appearances would be the same, the amount of blood and the length of time since the occurrence varying the picture. The treatment suggested for hypopyon is applicable here also. Cholesterine crystals are sometimes found in this part of the eye, generally in eyes the seat of old irido-choroiditis. The crystals may be seen heaped in a mass at the bottom of the chamber, or scattered over the posterior layer of the cornea, or, as in many instances, on the anterior surface of the iris. The shiny white and yellowish crystals give a beautiful appearance, especially when light is thrown on them. No treatment is called for, as this condition usually occurs in sightless eyes. Gummy and spongy exudations are also found in this space.

Entozoa of two varieties are found to inhabit this region, namely, the filaria and cysticercus. Various names are given to this parasite according to its location, as, Filaria oculi humani; F. equini; F. papillosa, and F. medinensis.

It is a specie of the Guinea thread-worm, but smaller; the variety found in the eye is known as F. papillosa. It has long been known that this parasite is occasionally found floating about in the anterior chamber of the horse's eye; though rare in this climate, it is by no means uncommon in India, where it is seen only during the cold months, not being observed before the beginning of October or later than the end of February.

The more constant the rain during the periodical rainy season, particularly toward its close, the more numerous the cases of filaria during the subsequent cold months.

It occurs chiefly in the low districts, being rare in the upper provinces, where the soil is drier. At Poonah usually twenty cases occur annually. One year thirty cases were seen; and at Ghazepoor, higher up, situated on dry soil, none were observed. During the rainy season the water is stored in large tanks for use during the subsequent dry season. In this water has been found a "tank-worm," by some thought to be a larvæ form of filaria, which, when taken into the system, develops and finally reaches its habitat in the aqueous humor.

The worm is very active, and swims about in the aqueous, sometimes going through the pupil to the posterior chamber. Usually one is found, but cases are on record where two have been seen in the same eye. The worm is about one inch long, equal in size to sewing-thread, white or darker colored, having a mouth, simple intestinal canal, uterus, and prominent anal aperture.

If the parasite is very active, the irritation of the eye is considerable, causing redness, turbidity of the aqueous, and in some cases keratitis, which, if not relieved by removal of the worm, will cause destruction of the eye.

These results may be prevented by extracting the animal, by paracentesis corneæ, the aqueous on escaping carrying the worm along.

Should it not come away at the first operation, after a day or so it may be repeated. A case is reported where the tapping the chamber so disturbed the animal that it died and became subsequently absorbed.

This worm has been seen in the horse's eye in Europe and in this country. In this city a number of cases have been operated on in the past twenty years; it is, however, a rarity in this climate.

It has been found in the gadus æglefinus (haddock) in the aqueous, and also in the human eye. When found in the latter it is called F. oculi humani. The symptoms of irritation are the same as in the horse's eye.

In some fishes they are so numerous in the crystalline lens as to render it turbid and opaque. They have been found in the human lens, between the capsule and its surface; a case is on record where one was found under the conjunctiva oculi and extracted.

*Cysticercus Cellulosæ.*—This hydatid, consisting of a globular vesicle with a slender neck, of which the end is a trifle enlarged, forming a head with projecting hooklets, has been found in the human eye, usuallyin the anterior chamber, sometimes in the back part of the eye. It is also found in the eye of the pig.

This parasite is taken into the human system by eating pork. Pigs affected by this are said to be measled, and their flesh is called called measly pork. The French term the affection ladrerie, while the Germans call these creatures fiunen.

About twenty cases have been reported of cysticercus in the human eye—the first by Soemmering, in 1829. The diagnosis is not difficult; the vesicle shows at times very decided movements, especially when the pupil is stimulated to contraction by the action of strong light, the head and neck of the animal being then stretched out and moved about. The vesicle may either be free in the chamber or be partly adherent to the iris or cornea. They grow rapidly at first from the size of a pin's head to be as large as a pea, in some cases larger. When they are in the anterior chamber it is proper practice to extract the vesicle entire, if possible, as the contents are irritating, and if ruptured might cause serious inflammation of the eye. They are also found in the orbit and vitreous humor. In the pig's eye they present the same appearance.

